# Protocol for *ex vivo* competition and sequencing of mycobacterium isolated from infected guinea pigs

**DOI:** 10.1016/j.xpro.2022.101804

**Published:** 2022-10-29

**Authors:** Saba Naz, Shruti Dabral, Dhiraj Kumar, Vinay Kumar Nandicoori

**Affiliations:** 1 Mycobacterial Signalling Lab, CSIR-Centre for Cellular and Molecular Biology, Habsiguda, Telangana 500007, India; 2 Signal Transduction Lab-I, National Institute of Immunology, Aruna Asaf Ali Marg, New Delhi, Delhi 110067, India; 3 Cellular Immunology Group, International Centre for Genetic Engineering and Biotechnology, New Delhi, Delhi 110067, India

**Keywords:** Genomics, Sequencing, Immunology, Microbiology, Model organisms

## Abstract

We describe steps for gDNA isolation from mycobacterium strains isolated from guinea pig lungs. We detail steps for infection of guinea pigs with *Mycobacterium tuberculosis*, followed by *in vitro* growth, gDNA isolation, and whole genome sequencing. We also describe an *ex vivo* competition experiment to determine the selective advantage of one strain over another. We include details for WGS and mutation spectrum analysis. The protocol can be used to identify mutations that arise in other pathogenic bacteria.

For complete details on the use and execution of this protocol, please refer to Naz et al. (2021).

## Highlights

• Steps for the guinea pig infection experiment with Mycobacterium
• Steps for genomic DNA isolation from *Mtb* grown *in vitro* and *in vivo*
• Steps for WGS and mutation spectrum analysis
• Optimized protocol for competition experiment *ex vivo*

## Abstract (editor highlights)

Publisher’s note: Undertaking any experimental protocol requires adherence to local institutional guidelines for laboratory safety and ethics.

## Graphical abstract

**Figure:**
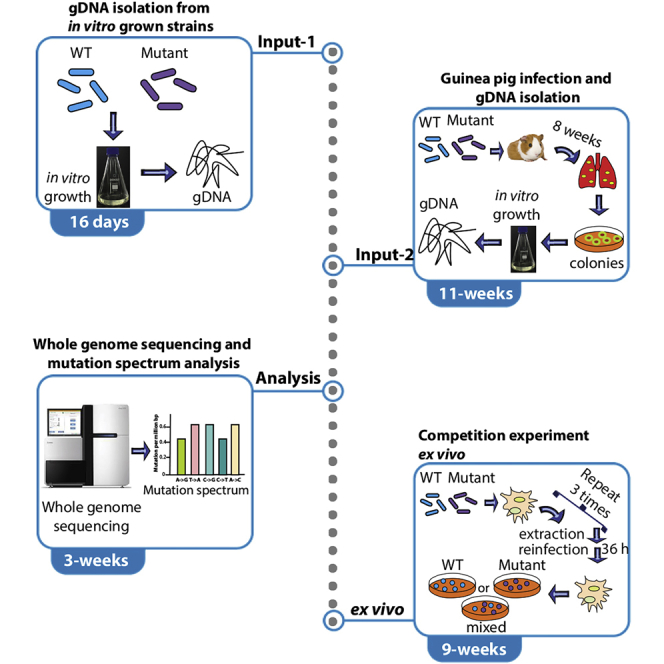

## Before you begin

*Mtb* encodes for ∼4000 genes. Mutations that would compromise/enhance the function of multiple genes such as DNA polymerase, DNA repair, and those involved in adaption to oxidative and nitrosative stress would impact genetic integrity. Reduced or increased acquisition of SNPs due to compromised or enhanced function of the above-described genes would, in turn, influence the survival capabilities of the strain. SNP identification can be performed in two different ways. 1. PCR amplification of known hotspot regions followed by Sanger’s sequencing. 2. Unbiased whole genome sequencing for identification of SNPs across the genome. The second methodology allows us to identify unknown SNPs that may play a role in survival/drug resistance. While the protocol is written from the context of DNA repair-deficient strains of *Mtb*, it can be used to evaluate any mutation affecting genome integrity. Furthermore, one can apply this methodology to other pathogenic bacterial species.

Mutations/ deletion of DNA repair genes impacts the repair process resulting in the accumulation of SNPs. While the influence of deleting a DNA repair gene may be limited when the cultures are grown *in vitro*, it is likely to have a significant impact when the bacilli encounter host stresses. We employed the guinea pig infection model to identify SNPs and determine the mutation spectrum. The Guinea pigs are outbred strains with a more robust immune response. Female Hartley guinea pigs were challenged with bacteria via the aerosol route. Genomic DNA from the bacterial colonies obtained from the guinea pig lungs were sequenced.

We also describe a competition experiment *ex vivo* wherein a selective advantage of one strain over another can be determined. WT and mutant strains independently and together (WT+ mutant at 1:1 ratio) were used for the infection experiment. 36 h post-infection, strains were extracted and used for reinfection of freshly seeded host cells. The process was repeated three times over to determine competitive advantage of one strain over the another.

### Ethical statement

*Mtb* is a BSL3 pathogen. All the experiments were performed in BSL3 or ABSL3 (animal BSL3) facilities. Institute Biosafety Committee has approved the execution of the project and standard operating procedures.

### Institutional permissions section

The Institute Animal Ethics Committee approved the animal experiment protocols. The approved protocol (IAEC#409/16) was used for performing the experiments as per the guidelines issued by the Committee for Control and Supervision of Experiments on Animals (CPCSEA), Government of India.

## Key resources table

**Table:** 

REAGENT or RESOURCE	SOURCE	IDENTIFIER
BBL thioglycollate medium brewer modified	Fisher Scientific	Cat No: 211716
BBL seven H11 agar base	Fisher Scientific	Cat No: 212203
Difco Middle brook 7H9 broth	Fisher Scientific	Cat No: 271310
Tween 80	Sigma-Aldrich	Cat No: P1754
Glycerol	Sigma-Aldrich	Cat No: G5516
Cycloheximide	Sigma-Aldrich	Cat No: C7698
PANTA mix	HiMedia	Cat No: FD260
Hyclone RPMI-1640 medium	Cytiva	Cat No: SH30255.01
Hyclone Fetal Bovine Serum	Cytiva	Cat No: SH30160.03
Phosphate-buffered saline (10×)	Cytiva	Cat No: SH30258.01
Kanamycin Monosulfate	Gold Biochem	Cat No: K-120-25
Hygromycin B Solution	HiMedia	Cat No: A015
Catalase	Sigma	Cat No: C1345
Agarose Special	HiMedia	Cat No: Mb002-5009
Sodium chloride	Sigma	Cat No: S3014
Oleic acid	Sigma	Cat No: O1008
Penstrep	Cytiva	Cat No: SV30010
Sodium dodecyl sulfate	Sigma	Cat No: L3771
DNeasy Blood & tissue kit	QIAGEN	Cat No: 69506
Qubit DS DNA HS Assay kit	Thermo Fisher Scientific	Cat No: Q32854
Petri dish 10 cm	Tarsons	Cat No: 462030
Cell Star 24 well plates	Greiner	Cat No: M8812
Hemocytometer	Merck	Cat No: BR717820
Albumin	Bio Basic Canada	Cat No: AD0023
Formaldehyde	Merck	Cat No: 1.94900.0521
Agencourt AMPure XP beads	Beckman Coulter	Cat No: A63880

**Experimental models: Organisms/strains**		

Balb/c mice (4–6 weeks old, both male and female can be used)	National Institute of Immunology small animal facility, New Delhi, India.	NA
Duncan Hartley guinea pigs (4–6 weeks old, both male and female can be used)	Lala Lajpat Rai University of Veterinary and Animal Sciences, Hisar, Haryana	NA

**Software and algorithms**		

FastQC	Andrews, 2010	https://www.bioinformatics.babraham.ac.uk/projects/fastqc/
bowtie2	Langmead and Salzberg, 2012	http://bowtie-bio.sourceforge.net/bowtie2/manual.shtml
Rv reference genome	Cole et al., 1998	https://www.ncbi.nlm.nih.gov/nuccore/NC_000962.3
Samtools	Li et al., 2009	http://www.htslib.org/
Qualimap	Okonechnikov et al., 2016	http://qualimap.conesalab.org/
VarScan software	Koboldt et al., 2012	http://varscan.sourceforge.net/
SnpEff toolbox	Cingolani et al., 2012	http://pcingola.github.io/SnpEff/
R studio latest version	Team, 2015	https://www.rstudio.com/products/rstudio/download/

## Materials and equipment

Heat inactivate FBS at 56°C for 30 min. Aliquot it and store at −20°C.

**Table:** Complete medium

	Final concentration	Amount
RPMI-1640	–	450 mL
Heat inactivated FBS	10%	50 mL
Pen-strep solution	1×	5 mL

Store the complete medium at 4°C.

**Table:** Genomic DNA lysis buffer

	Final concentration	Amount
Tris-Cl (pH=8)	20 mM	100 μL
Triton X-100	1.2%	60 μL
Lysozyme	2% (w/v)	100 mg
EDTA	2 mM	20 μL
Water		make up the volume to 5 mL

Aliquot the buffer in 2 mL Eppendorf tubes and store it at −20°C.

**Table:** ADC

	Final concentration	Amount
Albumin	5%	50 g
Dextrose	2%	20 g
Catalase	0.004%	40 mg
Sodium Chloride	0.85%	8.5 g
Water		1,000 mL

Filter sterilize and store it at 4°C.

**Table:** OADC

	Final concentration	Amount
Albumin	5%	50 g
Dextrose	2%	20 g
Catalase	0.004%	40 mg
Sodium Chloride	0.85%	8.5 g
Sodium oleate	4.4 mM	50 mL
Water		950 mL

Filter sterilize and store it at 4°C.

***Note:*** In 6 mL of oleic acid, add 12.5 mL NaOH (2 N) and dissolve in 500 mL.

Prepare aliquots of 50 mL sodium oleate and store it at −20°C.

**Table:** 7H9-medium

	Final concentration	Amount
Difco Middle brook 7H9 broth	0.47%	4.7 g
Tween 80	0.1%	1 mL
Glycerol	0.2%	2 mL
Water		1,000 mL

Autoclave and store it at 4°C.

**Table:** 7H11-medium

	Final concentration	Amount
BBL seven H11 agar base	0.98%	4.9 g
Glycerol	0.4%	2 mL
Water		450 mL

Autoclave and store it at 50°C. Once the temperature is ∼50°C, pour plates by adding one vial of PANTA, 50 mL OADC, and cycloheximide.

**Table:** Thioglycollate medium

	Final concentration	Amount
BBL thioglycollate medium	4%	4 g
Water		100 mL

Autoclave and store it at 4°C. It is recommended to store the freshly prepared thioglycollate at least for 20 days at 4°C for maturation and use it subsequently for injection.

**Table:** Strain storage medium

	Final concentration	Amount
OADC	2.64 mM	30 mL
50% Glycerol	20%	20 mL

Filter sterilize the storage medium and store it at 4°C.

Prepare 0.05% SDS in water. Filter sterilize using a 0.2 micron filter. Store it at 25°C.

Prepare 0.7% Agarose in 1×-TE buffer. Store it at 25°C.

## Step-by-step method details

### Guinea pig infection experiment

**Timing: 11 weeks for step 1–25**

**Timing: 4 weeks for step 26**

This method describes the aerosol infection experiment using guinea pig model of TB infection. Colonies obtained on the 7H11-OADC agar plates are selected for the genomic DNA (gDNA) isolation.

1. Procure the Female Hartley guinea pigs (weight 300–500 gm) from the authorized animal facility. For each strain, three guinea pigs are euthanized on day one post-infection, and six or seven guinea pigs are euthanized 8-week post-infection.

***Note:*** Male guinea pigs can be used for performing infection experiments. Female guinea pigs are preferred because they are easy to handle.

**CRITICAL:** Guinea pigs should not be treated with any antibiotics.

2. Transfer the guinea pigs randomly in the ventilated cages (n=2 per cage) in the aerosol challenge facility one week before infection to acclimatize them (Figure 1).

**Figure 1:**
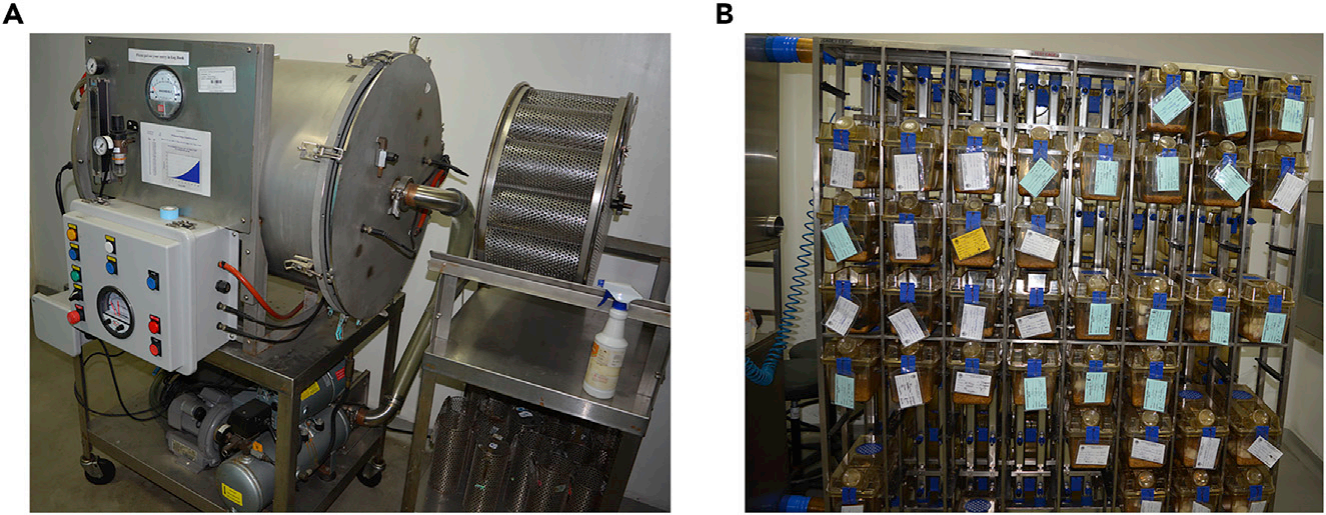
Guinea pig infection experiment setup in BSL3 facility. (A) Representative images of (A) the Madison chamber for aerosol infection. (B) The ventilated animal cages.
3. Thaw a glycerol stock of *Mtb*-H37Rv (laboratory strain of *Mtb*) and the mutant strain. Inoculate in 10 mL of 7H9-OADC medium. Grow the culture up to A_600_ ∼1.0 (generally takes seven days).
4. Inoculate both the strains independently in 20 mL of the 7H9-OADC medium at A_600_ ∼0.1 till A_600_ ∼0.8–1.0 (it takes ∼ 4 days, Table 1). Transfer the bacterial culture to a 50 mL Falcon tube. Pellet the bacterial cells at 3,220 × *g* for 10 min at 25°C and resuspend in 1 mL 1×-PBST_80_.

**Table 1:** Guinea pig infection experiment and WGS

S.No.	Experiment	Time	Additional suggestion
1.	Growth of *Mtb* strains in the 7H9-ADC broth	∼14 days	Take sufficient inoculum from a well grown colony.
2.	Preparation of 7H11-OADC/ 7H11-OADC -PANTA-cycloheximide containing plates.	Prepare plates one or two days before the experiment.	After pouring the plates, dry them so there will be no moisture. Stack the plates in an autoclave bag and incubate in 37°C incubator.
3.	Guinea pig lung isolation, preparation of lung homogenate and plating.	1 day	Guinea pigs should be cleaned with 70% ethanol prior to dissection.
4.	CFU enumeration	56 day post infection	Plates should be completely free of moisture and should contain PANTA and cycloheximide.
5.	Streaking of the colonies obtained from guinea pig lungs.	One day/per strain	Colonies will be fully grown in 14 days
6.	Growth of the streaked colonies in the 7H9-OADC medium	∼14 days	Fresh plates containing PANTA and cycloheximide should be prepared one day prior to usage.
7.	Isolation of gDNA	One day	Examine the quality of gDNA by resolving them on 0.7% agarose gel.
8.	Preparation of gDNA library	One day	Determine the quality of gDNA library using bioanalyzer / TAPE station.
9.	Analysis of WGS	21 days	Determine the SNPs accumulated with respect to the laboratory wild type strain.
5. Make up the volume to 20 mL after resuspension. Pellet the bacterial cells at 3,220 × *g* at 25°C and resuspend in 5 mL of Normal Saline.
6. Pass the bacterial cell suspension ten times using a 5 mL syringe containing a 26_1/2-_gauge needle.
7. Take the A_600_ of the culture.

**CRITICAL:** Taking A_600_ of bacterial cells is crucial; therefore, it should be done carefully.

8. Taking A_600_ of *Mtb* cells: Take three plastic cuvettes labeled them as Blank, Test and Confirm.
  a. Take 1 mL saline in the blank.
  b. Take 200 μL smooth culture+ 800 μL 1×-PBST_80_ in Test (i.e., culture is diluted 5×).
  c. Measure the absorbance at A_600_ using a spectrophotometer.
  d. Calculate the number of cells in the smooth culture by using the formula:
    0.6 A_600_ = 10^8^ cells per mL.
    For example: if the A_600_ is 0.3.
    Since the culture is diluted 5 times, the A_600_ of smooth culture is 0.3 × 5= 1.5.
    0.6 A_600_ = 10^8^ cells therefore 1.5 A_600_= 2.5 × 10^8^ cells.
    Hence 1 mL of smooth culture has 2.5 × 10^8^ cells.
  e. To confirm that the measured A_600_ and hence the number of cells calculated is correct, take that volume of cells in confirm cuvette that corresponds to 10^8^ cells and make up the volume to 1 mL.
    Example: Since 1 mL = 2.5 × 10^8^ cells.
    Therefore, 400 μL = 10^8^ cells (100 million).
    Add 600 μL 1×-PBST_80_ to make a volume of 1 mL. The A_600_ of this sample in confirm cuvette should be 0.6.
  f. Take 100 million (10^8^) bacterial cells for guinea pig infection in a 50 mL Falcon tube. Increase the volume to 15 mL using saline (the volume taken up by the nebulizer of the aerosol-generating machine is 15 mL).
    **CRITICAL:** Similar absorbance measurements should be performed for every strain used for the infection.
9. Randomly transfer the guinea pigs to the autoclaved aerosol cages (one cage per guinea pig) of the Madison Aerosol Exposure Chamber (Figure 1). Transfer the cell suspension (prepared in 8f) to the autoclaved nebulizer.
10. Female Hartley guinea pigs should be challenged via aerosol route for 20 min using the Madison chamber calibrated to implant 100 bacterial cells per guinea pig. (Chawla et al., 2014).
11. After the aerosol challenge, take out the guinea pigs and shift them to their respective cages. Label the cages properly.

***Note:*** Autoclave the aerosol cages and the nebulizer for the next round of infection with a different strain.

12. 24 h post-infection, randomly remove three guinea pigs (per strain) and euthanize them.
  a. Before dissection, clean the whole guinea pig using 70% ethanol.
  b. Take out the lungs after dissection.
13. Transfer the lungs to the saline-containing petri plates. Rinse the lungs properly in the saline to remove blood or other tissues.
14. Place the lungs containing petri plate next to a measurement scale and take a photograph. Transfer the lungs to the 50 mL Falcon tube containing 5 mL of saline.
15. Homogenize the lungs for 2 min at 10 × *g* using the tissue homogenizer. Give 2 min rest after each cycle.
16. Repeat step 15 until the sample is entirely homogenized.
17. Plate 500 μL of the lung homogenate to the 7H11-OADC-PANTA-cycloheximide containing square petri plates (Dimension- 120 mm × 120 mm). Dry the plates and stack them in a plastic bag. Incubate the plates at 37°C for three weeks.

***Note:*** Homogenizer probe used for preparing lungs lysate should be autoclaved.

**Figure 2:**
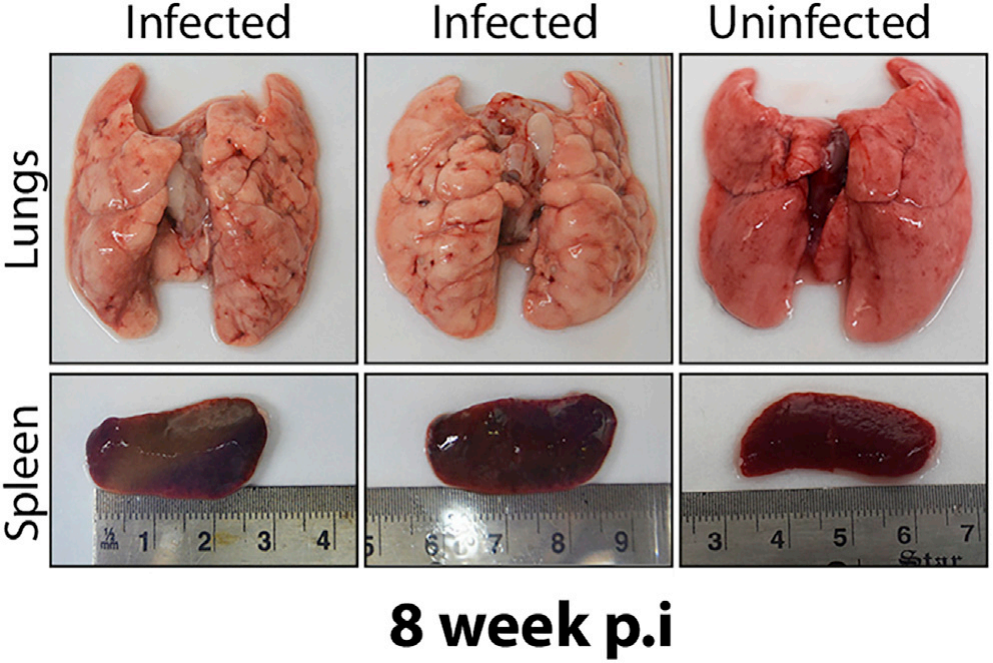
Representative image of lungs and spleen isolated from guinea pigs 8-week post-infection
20. Weigh lungs and spleen and note down their weight independently.

***Note:*** Uninfected control is required for determining the splenomegaly.

**Figure 3:**
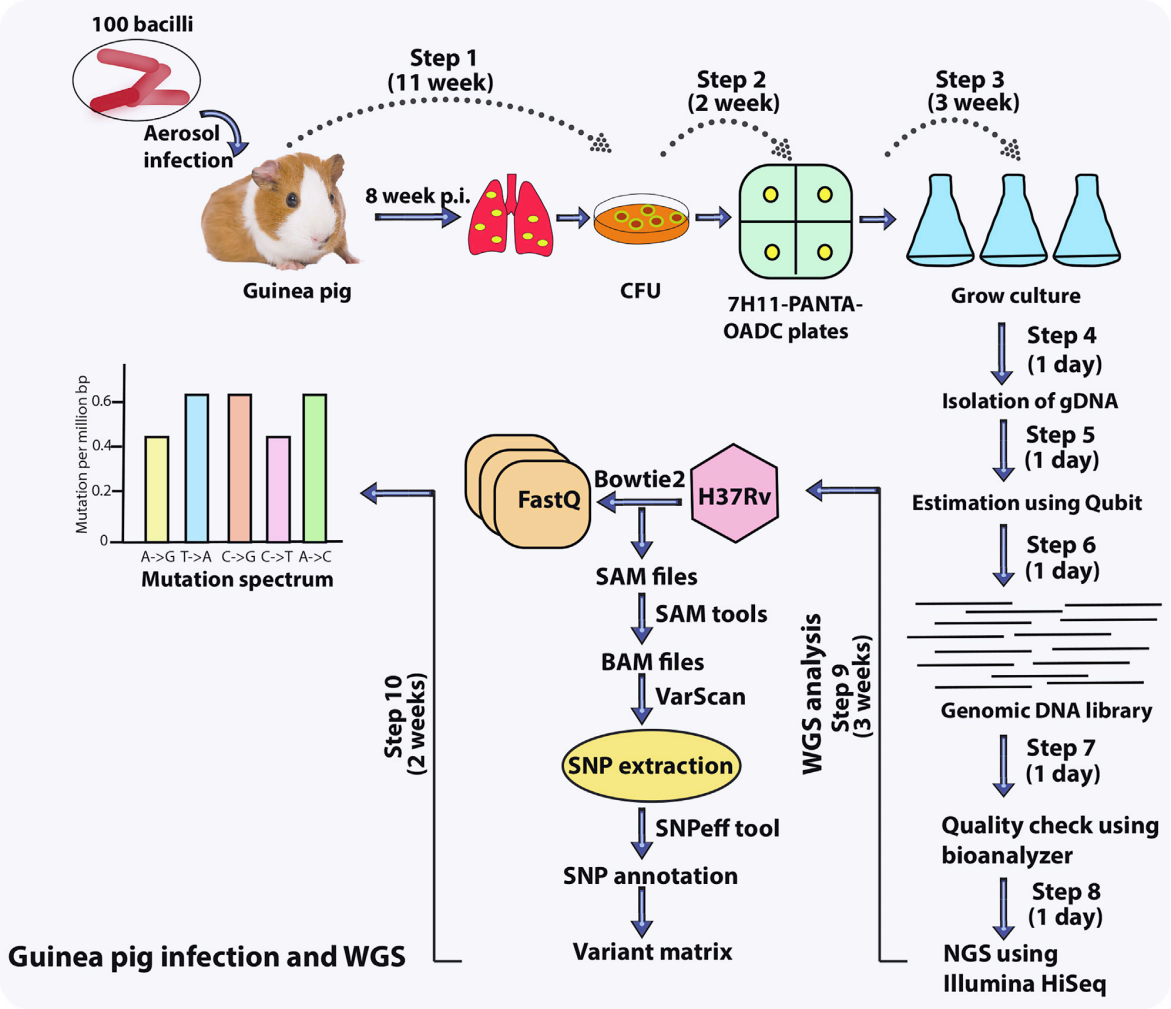
A schematic depicting the execution of the guinea pig infection experiment and the analysis of the mutation spectrum

18. At 8 weeks post-infection, take out guinea pigs (n=6 or 7 per strain). Perform the dissection. Isolate guinea pig lungs and spleen (repeat steps 12 and 14).
19. Take a photograph of the lungs and spleen along with a measurement scale (Figure 2).

21. Aliquot 5 mL of formalin in the tissue preserving vials. Cut a piece of lung and spleen and transfer it to the vial. Store the tissue-containing vial in the 4°C for ten days.
22. After 10 days, samples can be sent for H&E staining. The prepared slides can be sent to the expert for gross histopathology analysis.
23. After preserving the part of lung tissue, immediately transfer the lungs in the 50 mL Falcon tube containing 1×-PBST_80_. Homogenize the lungs and prepare lung homogenate (repeat steps 15 and 16).
24. Take 100 μL of the lung homogenate and prepare serial dilutions up to 10^−4^ in 1.5 mL sterile Eppendorf tube using 1×-PBST_80_.
25. Plate 100 μL of 10^−2^, 10^−3^, and 10^−4^ dilutions on the 7H11-OADC-PANTA-cycloheximide-containing plates. Plates should be thoroughly dried to prevent any moisture. Stack the dried plates in a plastic bag and store them at 37°C for 3 weeks (Figure 3).

**CRITICAL:** (a) The guinea pigs are not treated with anti-TB drugs before and after infection. (b) Fungal contamination is widespread in the lung’s homogenate-containing plates; therefore, it is recommended to add cycloheximide (final concentration, 40 μg/mL) in the 7H11-OADC-PANTA containing plates.

26. Once the bacterial colonies of WT and mutant strains start appearing on the plates, select them randomly (bacterial colonies may differ in size) and streak them on fresh 7H11-OADC-PANTA-cycloheximide-containing plates (Table 1). The number of bacterial colonies streaked on plates depends upon the experiment requirement. However, we suggest to streak at least ten bacterial colonies on 7H11-OADC-PANTA-cycloheximide-containing plates.
27. The number of bacterial colonies of each strain streaked on the 7H11-agar plates should be equally distributed amongst the guinea pigs. For example, if ten bacterial colonies are selected for the sequencing, it is recommended to streak at least two bacterial colonies from each guinea pig lung’s homogenate. This way, we maintain consistency and avoid bias.

**CRITICAL:** It is crucial to streak colonies on fresh 7H11-OADC-PANTA-cycloheximide-containing plates because at 37°C, most of the antibiotics start degrading, and the bacterial and fungal contamination increases significantly.

***Note:*** The number of colonies streaked on fresh 7H11-OADC-PANTA-cycloheximide plates depends on how many of them will be selected for sequencing. We recommend streaking at least 20 colonies for each strain.

28. When the streaks grow on plates, inoculate them in the 10 mL of 7H9-OADC medium. Grow the culture up to A_600_ ∼0.8 (∼ 7 days).
29. Subculture in fresh 30 mL 7H9- OADC medium at A_600_ ∼0.1. Grow the cultures to A_600_ ∼0.8–1.0 (∼4–5 days). Prepare two glycerol stocks of each strain in the strain storage medium. Preparation of glycerol stocks will be helpful when contamination occurs at the later stages, then, the glycerol stocks can be thawed and can be subsequently used for growing that particular colony.
30. Grow the antibiotic-sensitive laboratory H37Rv strain (at least three independent colonies) in a 7H9-OADC medium. Follow steps 28 and 29.

***Note:*** The passage number of all the strains used for sequencing should be the same to avoid accumulating other mutations that may arise due to the difference in the passage number.

**CRITICAL:** Keep monitoring the cultures for any contamination. Fungi contamination is widespread in the guinea pig lung homogenate. Fungi may start growing in the culture; if such culture is used to isolate genomic DNA, then the fungal genomic DNA contamination would significantly decrease the number of bacterial genome reads. In case of the fungi contamination discard the culture. Start with the new colony or the glycerol stock (steps 28 and 29).

### Genomic DNA isolation

**Timing: 6 h**

This method describes the gDNA isolation of the colonies obtained from guinea pig lungs for WGS.

31. Isolate gDNA using the Qiagen DNeasy Blood & tissue kit.
32. Pellet cells at 3,220 × *g* for 10 min at 25°C (step 23) and discard the supernatant. Wash the pellet with the 1× PBST_80_. Pellet the cells at 3,220 × *g* for 10 min at 25°C and discard the supernatant.
33. Resuspend the pellet in the 200 μL gDNA lysis buffer and transfer it to the 1.5 mL Eppendorf tube.
34. Incubate the sample at 37°C for 30 min. Add 25 μL of Proteinase K and 200 μL of AL lysis buffer (provided with the gDNA isolation kit). Incubate at 56°C for 30 min.
35. Add 200 μL of 100% ethanol and vortex for 10 s to ensure proper mixing. Do not heat the samples at 95°C; it will significantly increase the gDNA degradation.
36. Pellet the sample at 6,400 × *g* for 3 min to remove the cell debris.

**CRITICAL:** If one transfers the sample without performing step 35, the debris will stick to the column, and you will face difficulty during the washing steps.

37. Transfer the supernatant to the spin column provided with the kit and centrifuge at 4,830 × *g* for 1 min.
38. Discard the flow-through and add 500 μL of AW1 wash buffer. Centrifuge at 4,830 × *g* for 1 min. Add 500 μL of AW2 wash buffer and centrifuge at 4,830 × *g* for 1 min. Discard the flow-through and give a dry spin for 3 min at 6,400 × *g.*
39. Transfer the spin column to sterile Eppendorf and add 60 μL of elution buffer (Tris-Cl, pH=8). Incubate for 2 min and centrifuge at 6,400 × *g* for 2 min.

**CRITICAL:** It is essential to heat the elution buffer at 70°C before adding it to the spin column. Heating the elution buffer to 70°C increases the yield of gDNA.

**Figure 4:**
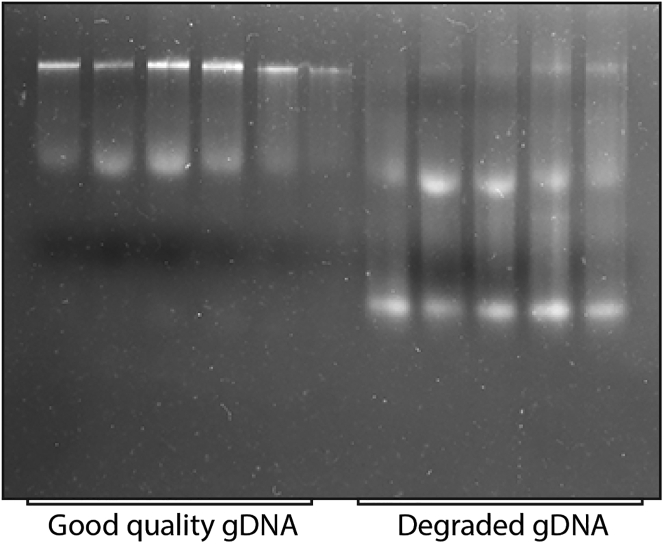
Agarose gel showing the good and poor quality of gDNA

40. Aliquot 5 μL of the sample in a separate Eppendorf tube and freeze the rest of the sample at −20°C.
41. Estimate the sample concentration using the HS dsDNA Qubit estimation kit. Follow the steps as given in the manufacturer’s protocol.
42. After estimating gDNA using a qubit, check the DNA integrity on an agarose gel. Load 100 ng of genomic DNA on the 0.7% agarose gel to check the quality (Figure 4).

**Pause point:** Isolated gDNA samples can be stored at −20°C until gDNA library preparation.

### Preparation of the genomic DNA library

**Timing: 8 h**

This method describes the preparation of gDNA library for WGS.

43. Qiagen QIAseq FX DNA library kit is used for gDNA library preparation.
44. The gDNA library preparation protocol is divided into three steps:
  a. fragmentation, End-repair, and A- addition,
  b. adapter ligation, and,
  c. PCR amplification of the library.
45. Take 100 ng of the genomic DNA in a PCR tube, and add 10 μL FX enzyme mix and 5 μL of FX buffer (provided with the kit) to make up the sample volume to 40 μL using nuclease-free water.
46. Incubate the reaction mix at 4°C for 1 min, 32°C for 6 min, 65°C for 30 min, and keep at 4°C for hold (perform this step in PCR machine, which is pre-heated lid at 70°C).
47. Add 5 μL of the DNA adapter to the fresh Eppendorf tube for adapter ligation.

***Note:*** Adapters are provided in the 96 well plate. Spinning the 96 well plate before taking the adapter DNA is advisable.

48. Transfer the sample to 5 μL adapter DNA containing an Eppendorf tube. Prepare ligation master mix by adding 20 μL ligase buffer, 10 μL DNA ligase, and 15 μL of nuclease-free water and add in the Eppendorf tube containing adapter DNA.
49. Incubate the mixture at 20°C for 15 min in a water bath. Immediately after the ligation, perform clean-up using Agencourt AMPure XP beads.
50. Add 80 μL of beads to the reaction mix, resuspend well using a 200 μL pipette tip, and incubate at RT for 5 min. Pellet beads on a magnetic stand, and discard the supernatant.
51. Wash beads thrice with 80% ethanol, and every time pellet on the magnetic stand (clean-up step).
52. Dry beads and elute the DNA library in 55 μL of elution buffer. Perform the second clean-up (as described in steps 50 and 51).
53. Finally, elute the DNA library in 23.5 μL of elution buffer.
54. Perform PCR amplification by mixing the library DNA (23.5 μL), primer mix (1.5 μL), and HiFi PCR master mix (25 μL) at cycling conditions,

**Table:** 

Steps	Temperature	Time	Cycles
Initial Denaturation	98°C	2 min	1
Denaturation	98°C	20 s	8 cycles
Annealing	55°C	30 s	
Extension	72°C	30 s	
Final extension	72°C	1 min	1
Hold	4°C	Forever

55. After performing PCR, clean up the library (described in steps 50 and 51).
56. Elute the purified library in 17 μL of elution buffer (Tris-Cl, pH=8). Aliquot 2 μL of prepared DNA library in a fresh Eppendorf and store 15 μL at −20°C.

**Pause point:** Library can be stored at −20°C until sequencing.

57. Check the quality and determine the concentration. Estimate the library using the Agilent Bioanalyzer. Follow the guidelines as per the manufacturer’s protocol.
58. The virtual image obtained from the bioanalyzer represents the ladder ranging from 35 to 10,380 bp. The prepared library should come under this ladder range. The electrophoresis file run summary shows the virtual gel image of 11 samples, which suggests that all the libraries are in the same ladder range (Figure 5).

**Figure 5:**
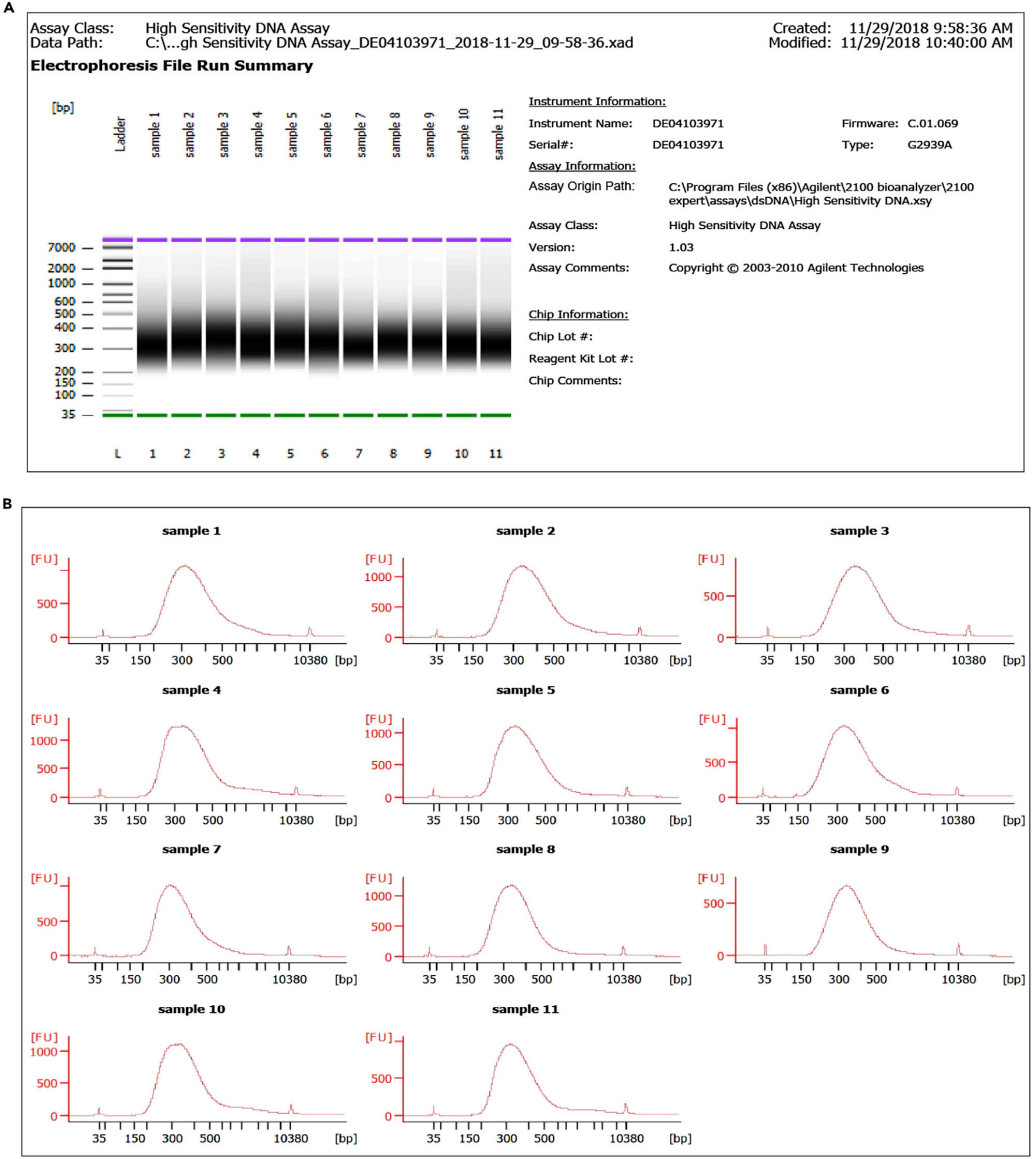
Quality check using a bioanalyzer (A) The virtual gel represents genomic DNA library. (B) Distribution of genomic DNA library and its concentration.
59. The distribution curve for each sample showed an average distribution of genomic DNA library around 350 bp. All the samples should pass the quality check value, and they should neither have primer nor adapter dimers.

### WGS analysis

**Timing: 3 weeks**

This method describes the WGS pipeline used for determining the mutation spectrum in the strains.

60. Generate the quality control report for all the RNA seq samples. We used Fastqc software.
  Download the software from https://www.bioinformatics.babraham.ac.uk/projects/fastqc/.

fastqc -f fastq /path/to/Rawsample/∗_1.fastq.gz

fastqc -f fastq /path/to/Rawsample/∗_2.fastq.gz

61. Align Fastq paired-end files on the H37Rv reference genome using bowtie2 software (Langmead and Salzberg, 2012).

[http://bowtie-bio.sourceforge.net/bowtie2/manual.shtml].

62. Download reference genome from “https://www.ncbi.nlm.nih.gov/nuccore/NC_000962.3 " and gtf file.

Create indexing file for MTB genome.

Run the command from your output directory.

mkdir /path/to/output_dir/index

cd /path/to/output_dir/index

bowtie-build -f H37Rv.fa Mtb

Make directory to store alignment results.

mkdir /path/to/Alignment_result/

bowtie2 --no-discordant --no-mixed --local -D 20 -R 2 -N 0 -L 32 -i L,0,1 -qx /path/to/output_dir/index/Mtb -1 /path/to/Rawsample/∗_1.fastq.gz -2 /path/to/Rawsample/∗_2.fastq.gz -S /path/to/Alignment_result/∗.sam

63. Convert generated SAM files into BAM files using Samtools (Li et al., 2009). [http://www.htslib.org/]

samtools view -Sb /path/to/Alignment_result/∗.sam > /path/to/Alignment_result/∗.bam

samtools sort /path/to/Alignment_result/∗.bam /path/to/Alignment_result/∗_sorted.bam

mkdir /path/to/mpileup

samtools mpileup -f /path/to/output_dir/index/H37Rv.fa /path/to/Alignment_result/∗_sorted.bam > /path/to/mpileup/∗_sorted.mpileup

64. Evaluate the alignment of the data and other quality parameters using Qualimap (Okonechnikov et al., 2016). [http://qualimap.conesalab.org/]

mkdir /path/to/quali

bash qualimap_v2.2.1/qualimap bamqc -bam/path/to/Alignment_result/∗_sorted.bam -gff /path/to/gtf/H37rv.gtf -outdir /path/to/quali/

65. Extract SNPs from BAM files using VarScan software (Koboldt et al., 2012). [http://varscan.sourceforge.net/]

mkdir /path/to/Varscan/

java -jar VarScan.v2.4.3.jar mpileup2snp /path/to/mpileup/∗_sorted.mpileup --min-coverage 30 --min-var-freq 0.5 --min-reads2 7 --p-value 0.01 --strand-filter 1 --output-vcf 1 > /path/to/Varscan/∗_sorted.vcf

66. Perform annotation of SNPs using SnpEff toolbox. [http://pcingola.github.io/SnpEff/]

mkdir /path/to/Varscan_Annotation/

java -Xmx40g -jar snpEff.jar Mycobacterium_tuberculosis_h37rv /path/to/Varscan/∗_sorted.vcf > /path/to/Varscan_Annotation/∗_vcf_annotated

mkdir /path/to/Tab_annotated/

cat /path/to/Varscan_Annotation/∗_vcf_annotated | ./vcfEffOnePerLine.pl | java -Xmx40g -jar SnpSift.jar extractFields -s \",\" -e \".\" - CHROM POS REF ALT \"ANN[∗].GENE\" \"ANN[∗].EFFECT\" \"ANN[∗].IMPACT\" \"ANN[∗].BIOTYPE\" \"ANN[∗].HGVS_P\" | grep -E -v \'(ˆ#|downstream_gene_variant|upstream_gene_variant)\' > /path/to/Tab_annotated/∗_tabdelim_annotation

mkdir /path/to/Final_annotation

awk \'{ print $5\"\\t\"$0 }\' /path/to/Tab_annotated/∗_tabdelim_annotation > /path/to/Final_annotation

awk -f vlookup_mod.awk Gene_annotation /path/to/Final_annotation/∗ > /path/to/Final_annotation/∗_final_annotation

67. Add blosum score to all final annotated file.

mkdir /path/to/blosum_results

cd /path/to/blosum_results

awk ' { print substr($10,3) } ' /path/to/Final_annotation/∗_final_annotation > amino

python blosum.py

awk ' { print $3 } ' /path/to/Final_annotation/∗_final_annotation > pos

paste pos score > ∗_final_annotation_blosum

68. Find the python script “blosum.py” in Data S1 (SupFile).
69. Combine all VCF files:“to create a matrix that includes chromosome position, a nucleotide position in the reference genome, the identified SNP in the newly sequenced genomes, SNP biotype, amino acid change, and respective genes/intergenic region” (Naz et al., 2021).
70. Laboratory *Mtb-H37Rv* strain was used a reference to identify unique SNPs.
71. Compare the SNPs identified in the laboratory grown bacteria versus *in vivo* isolated bacteria.
72. For mutation spectrum analysis (see Table 2), calculate;

**Table 2:** Mutation spectrum

Mutation	*H37Rv* (WT) sum	*Rv*Δ*dKO* (mutant) sum	Rv	*Rv*Δ*dKO* (mutant)
A->G	2	NA	0.041322	NA
C->A	6.	8	0.123966	0.2020202
C->G	4	9	0.082644	0.22727272
C->T	7	65	0.144628	1.641414141
G->A	1	71	0.020661	1.79292929
G->C	1	NA	0.02066	NA
G->T	2	NA	0.041322	NA
T->C	NA	8	NA	0.20202020
T->G	3	3	0.061983	0.0757575

The data are obtained from the Table S8 of the published article (Naz et al., 2021).

Mutation per million base pairs

= sum of all the mutations in all the sequenced samples / (divided by)

(4.4 × number of sequenced strain).

73. Install R studio latest version [https://www.rstudio.com/products/rstudio/download/] for further data plotting.

### Competition experiment *ex vivo*

**Timing: 5 days for step 74–81**

**Timing: 3 h for step 82**

We used primary cells, peritoneal macrophages, isolated one day prior to infection. We have observed that peritoneal macrophages show better results; therefore, we prefer performing experiments using peritoneal macrophages. However, these experiments can be performed in any other cell line such as THP1 or RAW 264.7. Since peritoneal macrophages are the primary cells, they can be used only up to 120 h post-infection. After 120 h post-infection, cells undergo apoptosis. It is crucial to have a continuous supply of peritoneal macrophages from the mouse during the reinfection stage.

74. **Isolation of peritoneal macrophages:** Inject 1 mL of thioglycollate in the peritoneal cavity of Balb/c mice, and on the 5^th^ day, isolate peritoneal macrophages. One can obtain approximately 8–10 × 10^6^ peritoneal macrophages per mouse (Zhang et al., 2008).
75. Aliquot 20 mL, 1× PBS (cell culture grade) in a Falcon tube. Euthanize mice and isolate peritoneal macrophages from the peritoneal cavity.

***Note:*** Both 1×-PBS and RPMI-1640 can be used to isolate the peritoneal macrophages. We prefer using 1× PBS (cell-culture grade for the isolation) as we can easily see the turbidity of cell suspension in the Falcon tubes compared to the RPMI-1640.

76. Pellet the peritoneal macrophages at 966 × *g* for 8 min.
77. Resuspend peritoneal macrophages in the RPMI-1640 medium containing 10% heat-inactivated Fetal Bovine Serum and 1× Pen-strep (complete medium). The resuspension volume depends on the number of mice used for the isolation of peritoneal macrophages. Generally, it is recommended to resuspend cells in 5 mL of complete medium if peritoneal macrophages are isolated from one mouse.
78. Take 10 μL of the cell suspension and count the number of cells using a hemocytometer. Count the peritoneal macrophages in all four quadrants and take an average.
  Example: No. of peritoneal macrophages per quadrant= 100, Therefore, total number = 100 × 10^4^ per mL.
79. Dilute the peritoneal macrophages in a complete medium such that each well contains 2.5 × 10^5^ cells in 500 μL complete medium per well.
80. Seed the peritoneal macrophages in 24 well cell culture plates and keep them in a cell culture incubator at 37°C. After 4 h, wash the peritoneal macrophages with 1× PBS to remove clumps and unadhered cells or tissue. Add 1 mL complete medium, incubate peritoneal macrophages at 37°C and perform infection the next day.
81. The next day an hour before performing infection, wash the seeded peritoneal macrophages with 1× PBS to remove the complete medium and add 500 μL of RPMI-1640 medium containing heat-inactivated 10% FBS.

***Note:*** This experiment requires the continuous passage of the strains in peritoneal macrophages. At every stage of reinfection, freshly isolated peritoneal macrophages are required.

82. **Preparation of bacterial cell suspension**: Grow the WT and mutant bacterial strain independently in 20 mL of 7H9-ADC medium up to A_600_ ∼0.8 (steps 3 and 4).

***Note:*** It is recommended to have different antibiotic markers for strains that are used for competition experiments.

83. Pellet the bacterial cells at 3,220 × *g* at 25°C and resuspend in 1 mL 1×-PBST_80_. Make up the volume to 20 mL after resuspension.
84. Pellet the bacterial cells at 3,220 × *g* at 25°C and resuspend in 1 mL of 1×-PBS. Transfer the suspension to 2 mL sterile Eppendorf tube.
85. Pass the bacterial cell suspension using a 1 mL syringe containing a 26_1/2_ gauge needle (10 times).
  A_600_ measurements:
  a. Set up the blank using 1×-PBS.
  b. Take 50 μL bacterial cell suspension and 950 μL of 1×-PBS. Take the absorbance.
  c. Calculate the number of bacterial cells as explained in step 8.
86. Dilute the bacterial cells in the RPMI-1640 medium containing heat-inactivated 10% FBS.
87. Perform infection at MOI=5 (5 bacterial cells per macrophage) (Figure 6).

**Figure 6:**
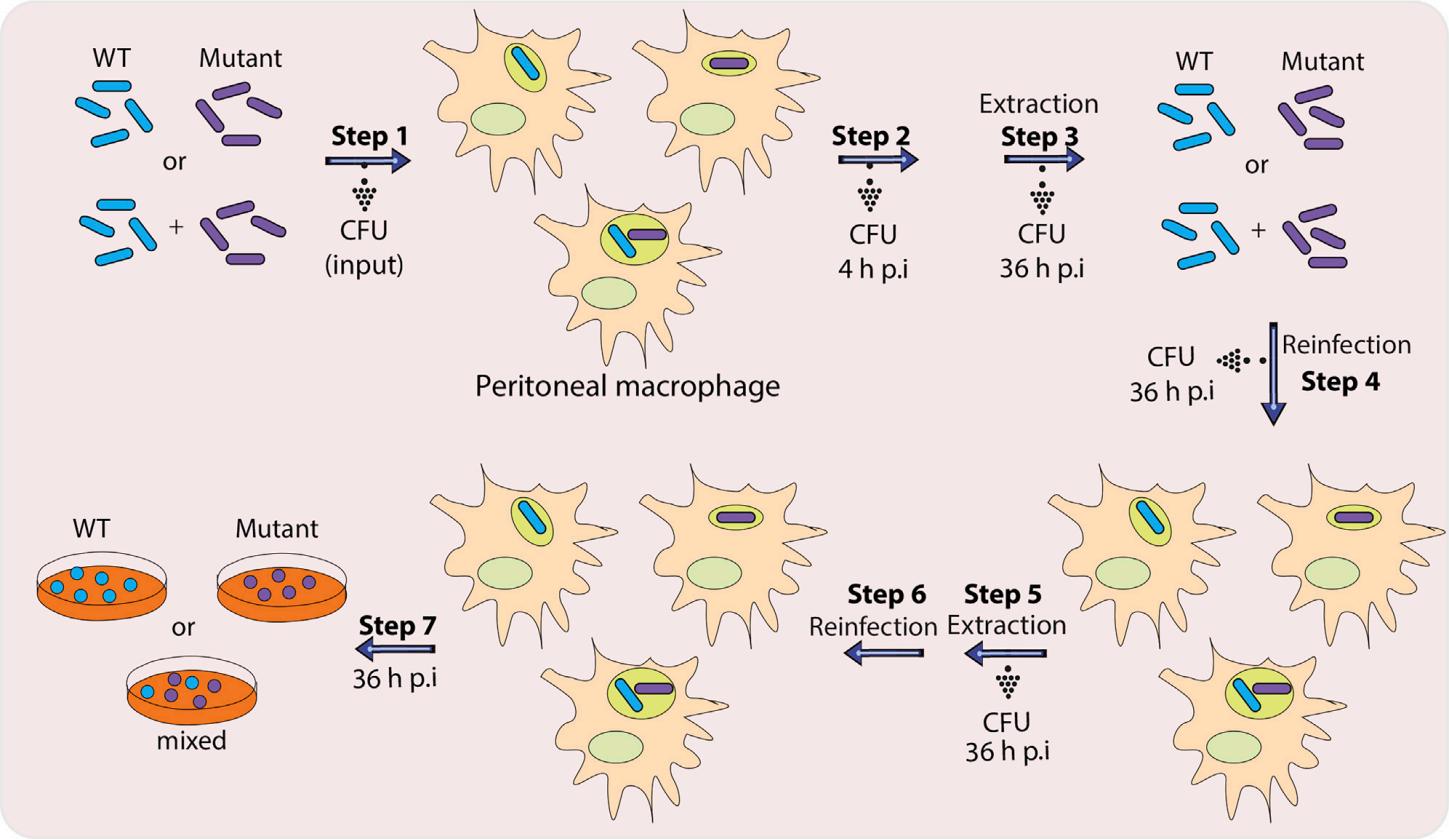
Schematic representing the competition experiment in peritoneal macrophages
88. Calculate the bacterial cells required for the infection experiment for both strains.

Peritoneal macrophages seeded= 0.25 × 10^6^ cells per well (step 79).

Bacterial cells = 2.5 × 10^5^ × 5 per well= 1.25 × 10^6^ bacterial cells per well for each strain.

89. Take sterile Eppendorf tubes or 15 mL Falcon tubes and label them as 1, 2, and 3. Add the required number of bacterial cells of each strain independently in the Eppendorf tubes labeled as 1, 2. Tube 3 is mixed infection, so add 0.625 × 10^6^ bacterial cells of strain 1 and 0.625 × 10^6^ bacterial cells of strain 2 cells. Mix them using the pipette.

**CRITICAL:** An aliquot of the finally diluted bacterial cells should be used for CFU plating on a plain 7H11-OADC agar plate to ensure an equal number of bacterial cells.

90. Add 100 μL bacterial suspension to each well containing peritoneal macrophages (prepared in step 80). Perform bacterial CFUs as described in steps 24 and 25.
91. Keep the infected peritoneal macrophages at 37°C for 4 h. Remove the medium and wash the infected peritoneal macrophages thrice using the 1×-PBS. Add 250 μL of 0.05% SDS in 4 wells, and in other wells, add complete medium.

***Note:*** 0.05% SDS is used for the lysis of the infected peritoneal macrophages; however, it cannot lyse the bacterial cells.

92. After 10 min incubation, take out 250 μL of 0.05% SDS in a sterile Eppendorf tube and prepare dilutions up to 10^−5^ in an Eppendorf tube. Take 100 μL of 10^−3^, 10^−4^, and 10^−5^ dilution and plate on a plain 7H11-OADC agar plate.
93. After drying the agar CFU plates, incubate them at 37°C for three weeks.
94. At 36 h post-infection, lyse the infected peritoneal macrophages and prepare dilutions using 50 μL infected peritoneal macrophages lysate as described in steps 24 and 25. Take 100 μL of 10^−3^, 10^−4^, and 10^−5^ dilution and plate on a plain 7H11-OADC agar plate. After drying the plates, incubate them at 37°C for three weeks.
95. Take 200 μL of remaining cell lysate obtained in step 91. Transfer it in a sterile 15 mL Falcon tube and add 10 mL of sterile 1× PBS. Pellet the bacterial cells at 3,220 × *g* for 20 min and decant the supernatant. Repeat the process twice to completely remove the traces of 0.05% SDS.
96. Finally, resuspend the bacterial cells in 100 μL of complete medium and add to freshly isolated peritoneal macrophages (see steps 90–95).
97. Repeat steps 90–95 twice.
98. At the final step, lyse the infected peritoneal macrophages in 50 μL of 0.05% SDS and plate 100 μL of 10^−1^ and 10^−2^ dilutions on a 7H11-OADC-containing plate. Incubate the plates at 37°C for three weeks.
99. Once bacterial colonies start growing on the plates, randomly select 100 bacterial colonies and spot them on different antibiotic-containing 7H11-OADC plates.

Example: If the hygromycin resistance marker is in strain 1 and the kanamycin marker in strain 2, spot them on both the plates.

100. Resuspend the bacterial colony (step 99) in 60 μL of 7H9-medium in an Eppendorf tube. Spot 30 μL each in hygromycin and kanamycin containing 7H11-OADC containing plates.
101. Incubate the plates at 37°C for three weeks.
102. Count the number of bacterial colonies on both the plates and plot the data as percent survival (Table 3).

**Table 3:** *Ex vivo* competition experiments

S. No.	Experiment	Time	Additional suggestion
1.	Growth of *Mtb* strains in the 7H9-ADC broth	∼14 days	
2.	Preparation of 7H11-OADC/ 7H11-OADC -hygromycin and kanamycin containing plates.	Plates should be prepared one or two days before experiment.	After pouring the plates, dry them so there will be no moisture. Stack the plates in an autoclave bag and store them in 37°C incubator.
3.	Injection of thioglycollate in the peritoneal cavity of the mouse	5 days	For a continuous supply of the peritoneal macrophages, inject the thioglycollate in the three batches of mice at the interval of one day.
4.	Isolation of peritoneal macrophages	1 day	Isolation of the peritoneal macrophages should be performed one day before infection.
5.	CFU enumeration post-infection	21 day post-infection	
6.	Spot the colonies obtained after the competition experiment on hygromycin and kanamycin containing 7H-11 OADC plates.	2–3 days	Randomly select 100 colonies and spot equal amounts on hygromycin and kanamycin containing 7H-11 OADC plates.
7.	CFU enumeration after growth on hygromycin and kanamycin containing 7H-11 OADC containing plates.	21 days	This step should be performed at every stage of the infection.

## Expected outcomes

A clean band on the agarose gel indicates good quality and quantity of genomic DNA. This sample can be used for the genomic DNA library preparation. WGS of the bacteria isolated from guinea pig lungs showed the spectrum of mutations that can be assessed by the mutation spectrum (Table 2). In a competition experiment, the CFU determination at every stage ensures the normalization of uptake of bacteria by the host cells (Table 3). Passage of the strains independently and *ex vivo* would determine whether one strain has a selective advantage over another.

## Limitations

It is essential to determine the CFU of the inoculum used for the *ex vivo* infection experiment at every stage to avoid any ambiguity in the analysis. Clean-up of adapters and primer dimers is a critical step in the library preparation. If the clean-up is not done well, then the library cannot be used for the sequencing (steps 49–51). It is essential to perform the *ex vivo* experiments with precautions. Any contamination in the peritoneal macrophages or bacterial CFU plates makes the whole experiment futile. One has to restart the experiment.

## Troubleshooting

### Problem 1

Genomic DNA yield is too low: It is possible that isolated genomic DNA obtained would be too low. It could be due to a few numbers of bacterial cells or due to insufficient lysis.

### Potential solution

Increase the number of bacterial cells. Divide the sample into three different Eppendorf. Follow step 35 and pool the supernatant and follow steps 33–39. This will increase the yield of the gDNA.

### Problem 2

DNA is degraded: DNA degradation and poor yield.

### Potential solution

It is essential to have a good quality of DNA to prepare a gDNA library. If the bacterial cells are heated after adding lysis buffer (step 34), this will cause DNA degradation. Therefore, do not heat the cells after lysis.

### Problem 3

Bacterial reads are less: The *Mtb* reads may show less percentage alignment after genome sequencing due to fewer bacterial reads.

### Potential solution

It could be due to the contamination with the gDNA preparation. It is most likely due to the fungal gDNA contamination. The genome of *Mtb* is 4.4 Mbp compared with the fungi. Therefore, during alignment, most reads may come from fungi that significantly reduce the *Mtb* reads. It is essential to pick up a single colony and monitor the culture at every stage to prevent contamination.

### Problem 4

Peritoneal macrophage yield is low: The yield of peritoneal macrophages may be less than expected.

### Potential solution

Low yield of peritoneal macrophages may be due to the use of freshly prepared thioglycollate. It is recommended to store the freshly prepared thioglycollate at least for 20 days at 4°C for maturation and use it subsequently for injection.

### Problem 5

Too much RBC in the isolated macrophages: RBC contamination may be very high while isolating the peritoneal macrophages (steps 74–76).

### Potential solution

Generally, the peritoneal macrophages form the white pellet. However, because of the RBC contamination, it becomes red. Resuspend the cell pellet in the 2 mL sterile 2 M sodium bicarbonate solution to remove the RBC contamination. Make up the volume to 50 mL. Incubate the cells for 5 min and centrifuge at 966 × *g* for 2 min. Resuspend the cells in complete medium and seed.

### Problem 6

Bacterial load is not equal at 4 h post-infection.

### Potential solution

It could be due to the bacteria’s unequal load used for infection or differential uptake. It is highly recommended to calculate the uptake of the bacteria. Calculate the uptake of bacteria by performing CFU of the bacterial suspension used for the infection (steps 24–25 and 88).

## Resource availability

### Lead contact

For information regarding reagents and resources will be directed to lead contact, Vinay Kumar Nandicoori (vinaykn@nii.ac.in and vinaykn@ccmb.res.in).

### Materials availability

This study does not require unique reagents.

## Data Availability

Codes used for analysis are given in the manuscript and in Data S1.

## Supplemental information

Data S1. Code used for generating the blosum score for identifying the effect of SNP analysis, related to step 68
