# Supplementary material for: Protocol for ex vivo competition and sequencing of mycobacterium isolated from infected guinea pigs
Source: STAR Protoc. 2022 Oct 29;3(4):101804. doi: 10.1016/j.xpro.2022.101804 (PMC9630790; doi:10.1016/j.xpro.2022.101804)
Supplement: Data S1. Code used for generating the blosum score for identifying the effect of SNP analysis, related to step 68 [file mmc1.pdf]

Supplementary File

SupFile: Code used for generating the blosum score for identifying the effect of SNP analysis (related to step 68).

For blosum.py script.

```
blosum62 = {"Cys":{"Cys":9, "Ser":-1, "Thr":-1, "Pro":-3, "Ala":0, "Gly":-3, "Asn":-3, "Asp":-3,
"Glu":-4, "Gln":-3, "His":-3, "Arg":-3, "Lys":-3, "Met":-1, "Ile":-1, "Leu":-1, "Val":-1, "Phe":-2,
"Tyr":-2, "Trp":-2},"Ser":{"Cys":-1,"Ser":4, "Thr":1, "Pro":-1, "Ala":1, "Gly":0, "Asn":1, "Asp":0,
"Glu":0, "Gln":0, "His":-1, "Arg":-1, "Lys":0, "Met":-1,"Ile":-2, "Leu":-2, "Val":-2, "Phe":-2, "Tyr":-2,
"Trp":-3},"Thr":{"Cys":-1,"Ser":1, "Thr":4, "Pro":1, "Ala":-1, "Gly":1, "Asn":0, "Asp":1, "Glu":0,
"Gln":0, "His":0, "Arg":-1, "Lys":0, "Met":-1, "Ile":-2, "Leu":-2, "Val":-2, "Phe":-2, "Tyr":-2, "Trp":-
3},"Pro":{"Cys":-3,"Ser":-1, "Thr":1, "Pro":7, "Ala":-1, "Gly":-2,"Asn":-1, "Asp":-1, "Glu":-1, "Gln":-
1, "His":-2, "Arg":-2, "Lys":-1, "Met":-2, "Ile":-3, "Leu":-3, "Val":-2, "Phe":-4, "Tyr":-3, "Trp":-
4},"Ala":{"Cys":0, "Ser":1, "Thr":-1, "Pro":-1, "Ala":4, "Gly":0, "Asn":-1, "Asp":-2, "Glu":-1, "Gln":-1,
"His":-2, "Arg":-1, "Lys":-1, "Met":-1, "Ile":-1, "Leu":-1, "Val":-2, "Phe":-2, "Tyr":-2, "Trp":-
3},"Gly":{"Cys":-3,"Ser":0, "Thr":1, "Pro":-2, "Ala":0, "Gly":6, "Asn":-2, "Asp":-1, "Glu":-2, "Gln":-2,
"His":-2, "Arg":-2, "Lys":-2, "Met":-3, "Ile":-4, "Leu":-4, "Val":0, "Phe":-3, "Tyr":-3, "Trp":-
2},"Asn":{"Cys":-3,"Ser":1, "Thr":0, "Pro":-2, "Ala":-2, "Gly":0, "Asn":6, "Asp":1, "Glu":0, "Gln":0,
"His":-1, "Arg":0, "Lys":0, "Met":-2, "Ile":-3, "Leu":-3, "Val":-3, "Phe":-3, "Tyr":-2, "Trp":-
4},"Asp":{"Cys":-3,"Ser":0, "Thr":1, "Pro":-1, "Ala":-2, "Gly":-1, "Asn":1, "Asp":6, "Glu":2, "Gln":0,
"His":-1, "Arg":-2, "Lys":-1, "Met":-3, "Ile":-3, "Leu":-4, "Val":-3, "Phe":-3, "Tyr":-3, "Trp":-
4},"Glu":{"Cys":-4,"Ser":0, "Thr":0, "Pro":-1, "Ala":-1, "Gly":-2, "Asn":0, "Asp":2, "Glu":5, "Gln":2,
"His":0, "Arg":0, "Lys":1, "Met":-2, "Ile":-3, "Leu":-3, "Val":-3, "Phe":-3, "Tyr":-2, "Trp":-
3},"Gln":{"Cys":-3,"Ser":0, "Thr":0, "Pro":-1, "Ala":-1, "Gly":-2, "Asn":0, "Asp":0, "Glu":2, "Gln":5,
"His":0, "Arg":1, "Lys":1, "Met":0, "Ile":-3, "Leu":-2, "Val":-2, "Phe":-3, "Tyr":-1, "Trp":-
2},"His":{"Cys":-3,"Ser":-1, "Thr":0, "Pro":-2, "Ala":-2, "Gly":-2, "Asn":1, "Asp":1, "Glu":0, "Gln":0,
"His":8, "Arg":0, "Lys":-1, "Met":-2, "Ile":-3, "Leu":-3, "Val":-2, "Phe":-1, "Tyr":2, "Trp":-
2},"Arg":{"Cys":-3,"Ser":-1, "Thr":-1, "Pro":-2, "Ala":-1, "Gly":-2, "Asn":0, "Asp":-2, "Glu":0, "Gln":1,
"His":0, "Arg":5, "Lys":2, "Met":-1, "Ile":-3, "Leu":-2, "Val":-3, "Phe":-3, "Tyr":-2, "Trp":-
3},"Lys":{"Cys":-3,"Ser":0, "Thr":0, "Pro":-1, "Ala":-1, "Gly":-2, "Asn":0, "Asp":-1, "Glu":1, "Gln":1,
"His":-1, "Arg":2, "Lys":5, "Met":-1, "Ile":-3, "Leu":-2, "Val":-3, "Phe":-3, "Tyr":-2, "Trp":-
3},"Met":{"Cys":-1,"Ser":-1, "Thr":-1, "Pro":-2, "Ala":-1, "Gly":-3, "Asn":-2, "Asp":-3, "Glu":-2,
"Gln":0, "His":-2, "Arg":-1, "Lys":-1, "Met":5, "Ile":1, "Leu":2, "Val":-2, "Phe":0, "Tyr":-1, "Trp":-
1},"Ile":{"Cys":-1,"Ser":-2, "Thr":-2, "Pro":-3, "Ala":-1, "Gly":-4, "Asn":-3, "Asp":-3, "Glu":-3, "Gln":-
3, "His":-3, "Arg":-3, "Lys":-3, "Met":1, "Ile":4, "Leu":2, "Val":1, "Phe":0, "Tyr":-1, "Trp":-
3},"Leu":{"Cys":-1,"Ser":-2, "Thr":-2, "Pro":-3, "Ala":-1, "Gly":-4, "Asn":-3, "Asp":-4, "Glu":-3,
"Gln":-2, "His":-3, "Arg":-2, "Lys":-2, "Met":2, "Ile":2, "Leu":4, "Val":3, "Phe":0, "Tyr":-1, "Trp":-
2},"Val":{"Cys":-1,"Ser":-2, "Thr":-2, "Pro":-2, "Ala":0, "Gly":-3, "Asn":-3, "Asp":-3, "Glu":-2, "Gln":-
2, "His":-3, "Arg":-3, "Lys":-2, "Met":1, "Ile":3, "Leu":1, "Val":4, "Phe":-1, "Tyr":-1, "Trp":-
3},"Phe":{"Cys":-2,"Ser":-2, "Thr":-2, "Pro":-4, "Ala":-2, "Gly":-3, "Asn":-3, "Asp":-3, "Glu":-3,
```

```
"Gln":-3, "His":-1, "Arg":-3, "Lys":-3, "Met":0, "Ile":0, "Leu":0, "Val":-1, "Phe":6, "Tyr":3, "Trp":1},"Tyr":{"Cys":-2,"Ser":-2, "Thr":-2, "Pro":-3, "Ala":-2, "Gly":-3, "Asn":-2, "Asp":-3, "Glu":-2, "Gln":-1, "His":2, "Arg":-2, "Lys":-2, "Met":-1, "Ile":-1, "Leu":-1, "Val":-1, "Phe":3, "Tyr":7, "Trp":2},"Trp":{"Cys":-2,"Ser":-3, "Thr":-3, "Pro":-4, "Ala":-3, "Gly":-2, "Asn":-4, "Asp":-4, "Glu":-3, "Gln":-2, "His":-2, "Arg":-3, "Lys":-3, "Met":-1, "Ile":-3, "Leu":-2, "Val":-3, "Phe":1, "Tyr":2, "Trp":11}
   }
   with open ("amino") as f:
     with open ("score","w") as g:
      for i in f.readlines():
        a=i[:3]
        b=i[-4:-1]
        try :
          score = blosum62 [a][b]
          z= str(score)
          g.write("%s\n" % (z))
        except KeyError :
          g.write("\n")
```
